# Associations of reallocating time between physical activity, sedentary behavior, and sleep with mental health among Chinese adolescents: an isotemporal substitution analysis

**DOI:** 10.1186/s12955-026-02611-y

**Published:** 2026-08-11

**Authors:** Jian Wu, Yongji Gao, Xuejun Chi, Haiyang Feng, Qiurui Yu, Yongjin Chen, Jieqiong Qin, Yinmei Yang

**Affiliations:** 1 College of Public Health, Zhengzhou University, Zhengzhou, China; 2 Shangqiu No.1 Senior Middle School, Shangqiu, China

**Keywords:** Adolescents, Mental health, Physical activity, Sedentary behavior, Sleep, Isotemporal substitution model

## Abstract

**Background:**

Depression and anxiety are important adolescent mental health concerns and may be linked to modifiable movement behaviors, including physical activity (PA), sedentary behavior (SB), and sleep. As mental health outcomes depend not only on time spent in specific behaviors but also on the trade-offs between them, this study examined the associations of reallocating 30 min between these behaviors with adolescent psychological symptoms.

**Methods:**

This cross-sectional study included 7,767 adolescents from Henan Province, Central China, in 2023. Daily PA, SB, and sleep duration were self-reported. Depressive symptoms were assessed using the 10-item Center for Epidemiologic Studies Depression Scale, and anxiety symptoms were measured with the 7-item Generalized Anxiety Disorder Scale. Isotemporal substitution models estimated the associations of 30-minute reallocations with depressive and anxiety symptoms. Interaction tests and subgroup analyses were conducted according to sleep status.

**Results:**

Replacing 30 min of SB with sleep or PA was associated with lower levels of depression (sleep, B = -0.47, 95%CI = -0.53~-0.41; PA, B = -0.12, 95%CI = -0.16~-0.08) and anxiety (sleep, B = -0.31, 95%CI = -0.36~-0.25; PA, B = -0.07, 95%CI = -0.10~-0.04). Reallocating PA to sleep also related to reduced depressive symptoms (B = -0.35, 95%CI = -0.42~-0.28) and anxiety symptoms (B = -0.24, 95%CI = -0.30~-0.18). Among adolescents with insufficient sleep, the associations were stronger when reallocating SB to sleep (for depression: B = -0.52, 95%CI = -0.64~-0.39; for anxiety: B = -0.40, 95%CI = -0.50~-0.28) or PA to sleep (for depression: B = -0.39, 95%CI = -0.50~-0.25; for anxiety: B = -0.32, 95%CI = -0.41~-0.19). Among those with sufficient sleep, reallocating SB to PA or sleep remained associated with lower depressive symptoms, but no significant association was observed for anxiety.

**Conclusions:**

Reallocating time toward sleep and PA was associated with reduced depressive and anxiety symptoms, particularly among adolescents with insufficient sleep. Accounting for sleep status may help advance understanding of the associations between movement behavior reallocations and adolescent mental health.

**Supplementary Information:**

The online version contains supplementary material available at 10.1186/s12955-026-02611-y.

## Introduction

Mental disorders constitute a major global public health challenge, frequently emerging during adolescence [1, 2]. An estimated 14% of adolescents aged 10 to 19 worldwide are affected, with depression and anxiety ranking as the most common conditions [3]. In China, the prevalence of adolescent mental health problems has shown a marked increase in recent years [4]. Given that internalizing problems in adolescence often persist into adulthood and impair long-term quality of life [5, 6], identifying modifiable risk factors is imperative for early prevention and mental health promotion.

Movement behaviors, including physical activity (PA), sedentary behavior (SB), and sleep, are important correlates of adolescent mental health [7, 8]. While regular PA has been associated with fewer depressive and anxiety symptoms [9], contemporary adolescents increasingly exhibit high levels of sedentariness, particularly screen-based SB, alongside chronic sleep restriction. These behaviors have been linked to adverse mental and physical health outcomes [10, 11]. This pattern is especially salient in the Chinese sociocultural context, where widespread device use and intense academic competition often prolong sedentary time and make sufficient sleep increasingly difficult to obtain [12]. Traditionally, research has examined PA, SB, and sleep as isolated predictors, overlooking their inherent temporal interdependence [13, 14]. Within a finite 24-hour day, movement behaviors are characterized by a zero-sum relationship, meaning that time invested in one activity inevitably detracts from another. Accordingly, an integrated analytical framework is imperative to examine how time reallocations among these behaviors correlates with mental health.

The isotemporal substitution model (ISM) addresses these behavioral trade-offs by estimating the associations that may arise when time allocated to one behavior is replaced with time for another while ensuring total time remains unchanged [15]. Previous studies among Western adolescents have reported associations between reallocating time from SB to sleep or PA and more favorable psychological outcomes [16, 17], but evidence remains sparse regarding Chinese adolescents [18, 19]. Furthermore, these associations may differ by an individual’s sleep status. According to the strength model of self-control, sleep provides the essential physiological basis for replenishing the finite energy required for emotional regulation [20]. Hence, adolescents suffering from chronic sleep deprivation may be more sensitive to restorative behavioral shifts.

Given the high co-occurrence of depressive and anxiety symptoms in youth [21, 22], this study applied ISM to examine both outcomes simultaneously. We aimed to evaluate the associations of time reallocations between PA, SB, and sleep with adolescent mental health, and to test whether these associations differed by sleep status. Subgroup analyses were conducted to further explore these relationships and provide evidence that may help inform more tailored movement behavior guidance.

## Methods

### Participants and procedures

This cross-sectional study was conducted between April and May 2023 using stratified random cluster sampling across six junior high schools in Henan Province, Central China. Within each selected school, 8 to 10 classes from grades 7 to 9 were sampled, and students in these classes were invited to participate in the study. Eligible participants included those who could understand and independently complete the questionnaire; conversely, adolescents were excluded if they had severe physical conditions limiting daily activities or cognitive impairments, or declined to participate.

**Fig. 1:**
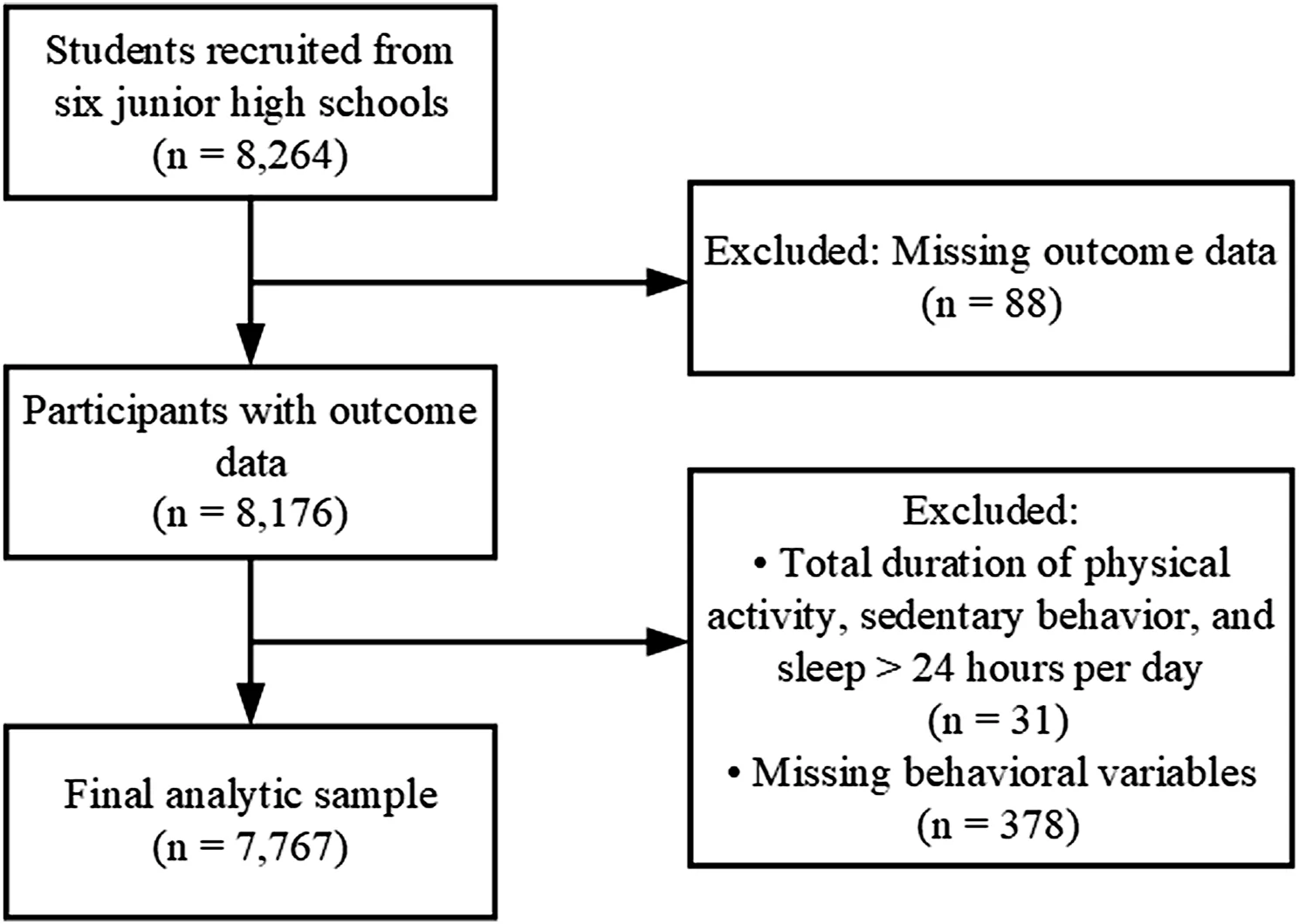
Flow diagram of analytic sample selection

Data collection was carried out during regular class sessions. Trained research assistants supervised the paper-and-pencil questionnaire and provided standardized instructions before completion. Voluntary participation and data confidentiality were emphasized. Of the 8,264 recruited students, 7,767 were included in the final analytic sample after excluding participants with missing outcome data, implausible or missing behavioral reports. Detailed information on analytic sample selection is illustrated in Fig. 1.

### Measures

#### Movement behaviors

Movement behaviors over the past week were assessed through three self-reported questions, asking participants to report their average daily duration of: (1) PA (“On average, how long did you spend on physical activity per day?”), (2) SB (“On average, how long did you spend sitting or reclining, excluding sleep per day?”), and (3) sleep (“On average, how long did you sleep per night, excluding time spent awake in bed?”). Sleep status was operationalized on the basis of nightly sleep duration, and insufficient sleep was defined as < 8 h per night, in accordance with recommendations for adolescents aged 13 to 17 [23–25].

#### Depression

Depressive symptoms were assessed using the 10-item Center for Epidemiologic Studies Depression Scale (CESD-10) [26]. Participants were asked to rate the frequency of each symptom on a 4-point Likert scale with responses from 0 (none or less than 1 day) to 3 (5–7 days). Items 4 and 9 were reverse-scored, and total scores ranged from 0 to 30. The Cronbach’s alpha was 0.851 in this study.

#### Anxiety

Anxiety symptoms were measured with the 7-item Generalized Anxiety Disorder Scale (GAD-7) [27]. Items were rated on a 4-point Likert scale from 0 (not at all) to 3 (nearly every day), with total scores ranging from 0 to 21. The Cronbach’s alpha was 0.906 in this study.

#### Covariates

Based on previous empirical evidence, sex and grade [28], residence [17], family socioeconomic status and family structure [29], academic performance [30], and academic pressure [31] were included as covariates. All covariates were assessed using a self-administered questionnaire.

### Statistical analysis

Descriptive statistics were performed to summarize participants’ characteristics, using means (M) and standard deviations (SD) for continuous variables and frequencies (n) with percentages (%) for categorical variables. Given the clustered sampling design, intraclass correlation coefficients (ICCs) were estimated from unconditional two-level random-intercept models using maximum likelihood estimation in Stata 18.0. The low ICCs for depression (0.037) and anxiety symptoms (0.043) indicated limited between-class variation [32].

In accordance with prior studies [33, 34], all behavioral variables were converted into 30-minute units to represent the estimated effect of a daily reallocation of 30 min. Three linear regression models were employed to examine associations of reallocating time between PA, SB, and sleep with depressive and anxiety symptoms.

First, the single-behavior model assessed the association of each movement behavior with mental health outcomes without adjustment for the remaining movement behaviors. Taking SB as an example, the model was expressed as:

Mental health score = (β1) SB + (β5) covariates.

Second, the partition model simultaneously included all three behaviors, thereby estimating the association of each behavior while mutually adjusting for the remaining two behaviors, without constraining total time. The model was expressed as:

Mental health score = (β1) SB + (β2) PA + (β3) sleep + (β5) covariates.

Finally, the ISM was used to estimate the associations of reallocating time between movement behaviors with mental health outcomes. A total time variable (the sum of PA, SB, and sleep) was included in the model together with two of the three behaviors, while the omitted behavior represented the behavior being replaced. In the following equation, the coefficients β2 and β3 represent the estimated differences in mental health scores when 30 min per day are reallocated from SB to PA and sleep, respectively, while holding total time constant.

Mental health score = (β2) PA + (β3) sleep + (β4) total time + (β5) covariates.

To examine whether the associations estimated by the ISM varied by sleep status, multiplicative interaction terms between sleep status and each isotemporal substitution term were included. Where significant interactions were identified (*P* < 0.05), subgroup analyses stratified by sufficient and insufficient sleep were conducted. All subsequent analyses were conducted using IBM SPSS Statistics 27.0, with statistical significance set at *P* < 0.05.

## Results

### Demographic and relevant characteristics

The characteristics of the study sample are summarized in Table 1. A total of 7,767 adolescents were included, comprising 4,140 (53.3%) males and 3,627 (46.7%) females. The mean age was 13.82 ± 0.93 years. Overall, 4,419 (56.89%) participants experienced insufficient sleep. The mean scores were 8.93 ± 5.75 for depression and 4.86 ± 4.76 for anxiety in the total sample. Among adolescents with sufficient sleep, the mean scores were 7.77 ± 5.24 for depression and 4.09 ± 4.19 for anxiety, compared with 9.81 ± 5.96 and 5.45 ± 5.08, respectively, among those with insufficient sleep. Additional distributional statistics, including medians, interquartile ranges, and ranges for sleep, PA, and SB, are presented in Supplementary Table S1.

**Table 1:** Demographic characteristics of the study sample (*n* = 7,767)

Variables	*n* (%) / M ± SD
**Age (years)**	13.82 ± 0.93
**Sex**	
Male	4,140 (53.30)
Female	3,627 (46.70)
**Grade**	
7	2,954 (38.03)
8	2,802 (36.08)
9	2,011 (25.89)
**Residence**	
Rural	2,396 (30.85)
Urban	5,371 (69.15)
**Family socioeconomic status**	
Poor	470 (6.05)
Average	5,923 (76.26)
Good	1,374 (17.69)
**Family structure**	
Intact	7,160 (92.18)
Non-intact	607 (7.82)
**Academic performance**	
Poor	1,962 (25.26)
Average	4,110 (52.92)
Good	1,695 (21.82)
**Academic pressure**	
Low	452 (5.82)
Average	4,272 (55.00)
Heavy	3,043 (39.18)
**Duration of movement behaviors (minutes)**	
Sleep	466.46 ± 56.91
PA	124.77 ± 95.66
SB	648.97 ± 187.90
**Sleep groups**	
Sufficient (≥ 8 h)	3,348 (43.11)
Insufficient (< 8 h)	4,419 (56.89)
**Mental health**	
Depressive symptoms	8.93 ± 5.75
Anxiety symptoms	4.86 ± 4.76

Note: PA = physical activity; SB = sedentary behavior; M = mean; SD = standard deviation

### Associations of time reallocations between movement behaviors with adolescent mental health

The single-behavior and partition models showed that greater sleep and PA time were associated with lower depressive and anxiety symptoms, whereas greater SB time was associated with higher symptom levels. Isotemporal substitution analyses revealed that reallocating 30 min from SB to sleep or PA was significantly associated with lower depression (sleep, B = -0.47, 95%CI = -0.53~-0.41; PA, B = -0.12, 95%CI = -0.16~-0.08) and anxiety (sleep, B = -0.31, 95%CI = -0.36~-0.25; PA, B = -0.07, 95%CI = -0.10~-0.04). Reallocating 30 min from PA to sleep was also associated with reduced depressive symptoms (B = -0.35, 95%CI = -0.42~-0.28) and anxiety symptoms (B = -0.24, 95%CI = -0.30~-0.18). Details are shown in Table 2.

**Table 2:** Single-behavior, partition, and isotemporal substitution models for movement behaviors and adolescent mental health

	Replacement behaviors								
	Sleep B (SE)	95%CI	*P*	PA B (SE)	95%CI	*P*	SB B (SE)	95%CI	*P*
**Depressive symptoms**									
Single behavior	-0.44(0.03)	-0.50, -0.38	< 0.001	-0.07(0.02)	-0.11, -0.03	< 0.001	0.05(0.01)	0.03, 0.07	< 0.001
Partition	-0.46(0.03)	-0.52, -0.39	< 0.001	-0.10(0.02)	-0.14, -0.07	< 0.001	0.01(0.01)	-0.01, 0.03	0.159
Isotemporal substitution									
Sleep	-	-	-	0.35(0.04)	0.28, 0.42	< 0.001	0.47(0.03)	0.41, 0.53	< 0.001
PA	-0.35(0.04)	-0.42, -0.28	< 0.001	-	-	-	0.12(0.02)	0.08, 0.16	< 0.001
SB	-0.47(0.03)	-0.53, -0.41	< 0.001	-0.12(0.02)	-0.16, -0.08	< 0.001	-	-	-
**Anxiety symptoms**									
Single behavior	-0.29(0.03)	-0.34, -0.23	< 0.001	-0.04(0.02)	-0.07, -0.01	0.018	0.03(0.01)	0.02, 0.05	< 0.001
Partition	-0.30(0.03)	-0.35, -0.24	< 0.001	-0.06(0.02)	-0.09, -0.03	< 0.001	0.01(0.01)	-0.01, 0.03	0.294
Isotemporal substitution									
Sleep	-	-	-	0.24(0.03)	0.18, 0.30	< 0.001	0.31(0.03)	0.25, 0.36	< 0.001
PA	-0.24(0.03)	-0.30, -0.18	< 0.001	-	-	-	0.07(0.02)	0.04, 0.10	< 0.001
SB	-0.31(0.03)	-0.36, -0.25	< 0.001	-0.07(0.02)	-0.10, -0.04	< 0.001	-	-	-

Note: PA = physical activity; SB = sedentary behavior; B = unstandardized regression coefficient; SE = standard error; CI = confidence interval. All models adjusted for grade, sex, residence, family socioeconomic status, family structure, academic performance and academic pressure

### Associations of time reallocations between movement behaviors with adolescent mental health by sleep status

Interaction tests presented in Table 3 suggested a moderating role of sleep status. Significant interactions were observed for reallocating SB to sleep (for depression: B = 0.31, 95%CI = 0.12 ~ 0.52; for anxiety: B = 0.32, 95%CI = 0.18 ~ 0.52) and PA to sleep (for depression: B = 0.26, 95%CI = 0.10 ~ 0.50; for anxiety: B = 0.28, 95%CI = 0.16 ~ 0.50), indicating that the associations were stronger among adolescents with insufficient sleep. The interaction for reallocating SB to PA was only marginally significant.

**Table 3:** Interaction effects between time reallocations and sleep status on adolescent mental health

Mental health	Interaction term	B (SE)	95%CI	*P*
**Depressive symptoms**				
	(SB→Sleep) × Sleep Status	0.31(0.10)	0.12, 0.52	0.002
	(SB→PA) × Sleep Status	0.07(0.04)	−0.01, 0.15	0.090
	(PA→Sleep) × Sleep Status	0.26(0.10)	0.10, 0.50	0.003
**Anxiety symptoms**				
	(SB→Sleep) × Sleep Status	0.32(0.09)	0.18, 0.52	< 0.001
	(SB→PA) × Sleep Status	0.06(0.04)	−0.01, 0.13	0.077
	(PA→Sleep) × Sleep Status	0.28(0.09)	0.16, 0.50	< 0.001

Note: PA = physical activity; SB = sedentary behavior; B = unstandardized regression coefficient; SE = standard error; CI = confidence interval. SB→Sleep denotes the reallocation of 30 min from SB to sleep. Sleep status was dummy-coded as 0 = insufficient sleep (reference group) and 1 = sufficient sleep. All models were adjusted for grade, sex, residence, family socioeconomic status, family structure, academic performance and academic pressure

Table 4 shows the subgroup results. Among adolescents with insufficient sleep, substituting 30 min of SB with sleep or PA was associated with reductions in depressive symptoms (sleep, B = -0.52, 95%CI = -0.64~-0.39; PA, B = -0.14, 95%CI = -0.19~-0.09) and anxiety symptoms (sleep, B = -0.40, 95%CI = -0.50~-0.28; PA, B = -0.09, 95%CI = -0.13~-0.05). Reallocating PA to sleep was also linked to fewer depression (B = -0.39, 95%CI = -0.50~-0.25) and anxiety symptoms (B = -0.32, 95%CI = -0.41~-0.19). In contrast, these associations were attenuated among adolescents with sufficient sleep. Replacing SB with sleep (B = -0.21, 95%CI = -0.36~-0.05) or PA (B = -0.08, 95%CI = -0.14~-0.02) remained associated with lower depressive symptoms in this group, whereas no significant associations with anxiety were observed.

**Table 4:** Single-behavior, partition, and isotemporal substitution models for movement behaviors and adolescent mental health stratified by sleep status

	Replacement behaviors								
	Sleep B (SE)	95%CI	*P*	PA B (SE)	95%CI	*P*	SB B (SE)	95%CI	*P*
**Depressive symptoms (Sufficient sleep)**									
Single behavior	-0.20(0.08)	-0.35, -0.04	0.013	-0.07(0.03)	-0.13, -0.01	0.028	0.03(0.02)	0.01, 0.06	0.039
Partition	-0.19(0.08)	-0.34, -0.03	0.017	-0.06(0.03)	-0.12, 0.01	0.066	0.02(0.02)	-0.01, 0.05	0.214
Isotemporal substitution									
Sleep	-	-	-	0.13(0.08)	-0.04, 0.29	0.124	0.21(0.08)	0.05, 0.36	0.008
PA	-0.13(0.08)	-0.29, 0.04	0.124	-	-	-	0.08(0.03)	0.02, 0.14	0.014
SB	-0.21(0.08)	-0.36, -0.05	0.008	-0.08(0.03)	-0.14, -0.02	0.014	-	-	-
**Depressive symptoms (Insufficient sleep)**									
Single behavior	-0.43(0.06)	-0.56, -0.31	< 0.001	-0.09(0.02)	-0.14, -0.05	< 0.001	0.03(0.01)	0.01, 0.06	0.007
Partition	-0.50(0.06)	-0.63, -0.38	< 0.001	-0.13(0.02)	-0.18, -0.08	< 0.001	0.01(0.01)	-0.01, 0.04	0.396
Isotemporal substitution									
Sleep	-	-	-	0.39(0.06)	0.25, 0.50	< 0.001	0.52(0.06)	0.39, 0.64	< 0.001
PA	-0.39(0.06)	-0.50, -0.25	< 0.001	-	-	-	0.14(0.03)	0.09, 0.19	< 0.001
SB	-0.52(0.06)	-0.64, -0.39	< 0.001	-0.14(0.03)	-0.19, -0.09	< 0.001	-	-	-
**Anxiety symptoms (Sufficient sleep)**									
Single behavior	-0.07(0.07)	-0.20, 0.06	0.264	-0.03(0.03)	-0.08, 0.02	0.243	0.01(0.01)	-0.02, 0.03	0.515
Partition	-0.07(0.07)	-0.20, 0.06	0.265	-0.03(0.03)	-0.08, 0.02	0.274	0.01(0.01)	-0.02, 0.03	0.828
Isotemporal substitution									
Sleep	-	-	-	0.04(0.07)	-0.09, 0.18	0.533	0.08(0.07)	-0.05, 0.20	0.246
PA	-0.04(0.07)	-0.18, 0.09	0.533	-	-	-	0.03(0.03)	-0.02, 0.08	0.224
SB	-0.08(0.07)	-0.20, 0.05	0.246	-0.03(0.03)	-0.08, 0.02	0.224	-	-	-
**Anxiety symptoms (Insufficient sleep)**									
Single behavior	-0.34(0.06)	-0.45, -0.23	< 0.001	-0.05(0.02)	-0.09, -0.01	0.012	0.03(0.01)	0.01, 0.05	0.011
Partition	-0.38(0.06)	-0.49, -0.27	< 0.001	-0.08(0.02)	-0.12, -0.04	< 0.001	0.01(0.01)	-0.01, 0.04	0.262
Isotemporal substitution									
Sleep	-	-	-	0.32(0.06)	0.19, 0.41	< 0.001	0.40(0.06)	0.28, 0.50	< 0.001
PA	-0.32(0.06)	-0.41, -0.19	< 0.001	-	-	-	0.09(0.02)	0.05, 0.13	< 0.001
SB	-0.40(0.06)	-0.50, -0.28	< 0.001	-0.09(0.02)	-0.13, -0.05	< 0.001	-	-	-

Note: PA = physical activity; SB = sedentary behavior; B = unstandardized regression coefficient; SE = standard error; CI = confidence interval. All models adjusted for grade, sex, residence, family socioeconomic status, family structure, academic performance and academic pressure

## Discussion

To our knowledge, this study constitutes the first attempt to integrate ISM with interaction analyses to examine whether sleep status acts as a moderator in the associations between time reallocations and mental health among Chinese adolescents. By extending traditional single-behavior analyses, ISM provides a more nuanced description of how time trade-offs across PA, SB, and sleep relate to depressive and anxiety symptoms. In the total sample, reallocating 30 min from SB to sleep or PA, or from PA to sleep, correlated with reduced depressive and anxiety symptoms. These associations were more pronounced among adolescents with insufficient sleep, whereas those with sufficient sleep showed weaker or non-significant associations. This pattern is consistent with recent evidence that favorable mental health profiles in youth typically emerge when high PA, low SB, and sufficient sleep coexist [35].

The associations observed when reallocating time from SB to PA or sleep align with the displacement hypothesis [36], and with recent movement behavior studies showing that such reallocations are associated with more favorable mental health outcomes [17, 37]. SB, especially screen-based SB common among adolescents, has been linked to depressive and anxiety symptoms [38]. The extant literature suggests that greater screen-based SB may coincide with less face-to-face social interaction and social support [39], as well as reduced time available for sleep and PA [40]. Conversely, PA has been associated with fewer psychiatric symptoms [41, 42]. Possible explanations proposed in previous studies include pathways related to social interaction, self-efficacy, and circadian regulation [43, 44]. Sleep also plays a pivotal role in emotional well-being, with recent evidence indicating that sufficient sleep may be associated with better neuroendocrine function and emotional regulation [45].

The interaction analyses suggested that the associations between time reallocations and mental health differed by sleep status. The association of reallocating time from SB to sleep or PA with lower depressive and anxiety symptoms was stronger among adolescents with insufficient sleep. This pattern may partly reflect their higher symptom burden and the close relationship between sleep debt and emotional difficulties [46]. In contrast, among adolescents with sufficient sleep, reallocating SB to sleep or PA showed weaker associations with depressive symptoms but no significant associations with anxiety. This may be related to the relatively lower and more stable levels of internalizing symptoms in this group. It is also possible that depressive and anxiety symptoms differ in their sensitivity to changes in daily movement behaviors. Prior research suggests that depressive symptoms may be more closely tied to fatigue, low energy, and reduced engagement in daily activities, whereas anxiety is more strongly characterized by cognitive worry and pre-sleep arousal [47]. Accordingly, among adolescents who already obtain sufficient sleep, replacing SB with other behaviors may still manifest in changes to depressive symptoms, yet is less likely to correspond to detectable shifts in anxiety.

A key finding was that reallocating PA to sleep was associated with lower depressive and anxiety symptoms among adolescents with insufficient sleep, whereas no such association was observed in the sufficient sleep group. This finding further suggests that sleep may be the limiting resource in adolescents who have inadequate sleep. For them, shifting time from PA to sleep may more directly address sleep debt and the accompanying emotional distress, whereas such a reallocation may offer diminished additional value once sufficient sleep has already been secured. This interpretation aligns with evidence showing that associations between replacing PA with sleep and lower levels of anxiety and depression may differ by sleep sufficiency [16]. These findings also support prior studies suggesting that the association between sleep duration and mental health may be non-linear, with additional sleep beyond recommended levels showing no further favorable associations [48].

These findings may have implications for adolescent mental health promotion and quality of life enhancement. When evaluating lifestyle-related mental health risk, school health services and family counseling may benefit from screening sleep status and tailoring guidance accordingly rather than adopting a one-size-fits-all approach. For adolescents with insufficient sleep, greater attention may be given to sleep restoration, alongside efforts to reduce SB and promote PA. Policies and school-based health promotion programs that support regular sleep schedules, limit late-evening screen use, and reduce unnecessary time pressure may help create a more supportive environment for balanced movement behavioral patterns and adolescent well-being.

### Limitations and future directions

Several limitations warrant consideration. First, the cross-sectional design precludes causal inferences. Moreover, the ISM focused on hypothetical reallocations among PA, SB, and sleep, not on the overall 24-hour composition, which may not fully reflect real-world behavioral substitutions. Although several associations were statistically significant, the relatively small magnitude of the estimated differences requires cautious interpretation regarding their practical implications. Future studies should collect complete 24-hour data, apply compositional analyses, and use longitudinal or experimental designs to clarify these associations. Second, although feasible and cost-effective, self-reported single-item measures are subject to recall bias and measurement error. Future research should incorporate objective assessments (e.g., actigraphy, polysomnography) and more comprehensive measures. Third, sleep-related measures were limited to nightly sleep duration; midday napping, sleep quality and subjective sleep satisfaction were not assessed. Future studies should incorporate multidimensional sleep measures. Finally, as the sample was limited to Henan Province, geographically broader replication is required.

## Conclusions

Reallocating 30 min of SB to sleep or PA, and PA to sleep, were associated with reduced depressive and anxiety symptoms among Chinese adolescents. These associations were stronger among adolescents with insufficient sleep, whereas those with sufficient sleep showed weaker associations with depressive symptoms and no significant links to anxiety. These findings suggest that sleep status may moderate how daily time reallocations relate to adolescent mental health. Integrating sleep status into the formulation of movement behavior guidelines could facilitate more tailored adolescent health promotion efforts within both school and family contexts.

## Supplementary Information

Below is the link to the electronic supplementary material.

Supplementary Material 1

## Data Availability

The data are available from the corresponding author on reasonable request.

## Abbreviations

PA: Physical activity
SB: Sedentary behavior
ISM: Isotemporal substitution model
CESD-10: 10-item Center for Epidemiologic Studies Depression Scale
GAD-7: 7-item Generalized Anxiety Disorder Scale
M: Mean
SD: Standard deviation
SE: Standard error
CI: Confidence interval
ICCs: Intraclass correlation coefficients
