# Supplementary material for: Associations of reallocating time between physical activity, sedentary behavior, and sleep with mental health among Chinese adolescents: an isotemporal substitution analysis
Source: Health Qual Life Outcomes. 2026 Aug 11;24:107. doi: 10.1186/s12955-026-02611-y (PMC13499305; doi:10.1186/s12955-026-02611-y)
Supplement: Supplementary file 1 [file 12955_2026_2611_MOESM1_ESM.docx]

**Supplementary Table S1** Additional Distributional Statistics for movement behaviors (minutes)

| **Variables** | **Medians (IQRs)** | **Ranges** |
| --- | --- | --- |
| Sleep | 469 (433–502) | 154–793 |
| PA | 100 (60–150) | 5–788 |
| SB | 660 (540–780) | 30–1140 |

Note: PA = physical activity; SB = sedentary behavior; IQRs = interquartile ranges.
